# Parity: A key measure of confounding in data-linkage studies of outcomes after medically assisted reproduction

**DOI:** 10.23889/ijpds.v5i1.1119

**Published:** 2020-03-16

**Authors:** GM Chambers, CA Venetis, LR Jorm, EP Stavrou, CM Vajdic

**Affiliations:** 1 Centre for Big Data Research in Health, University of New South Wales Sydney, NSW, Australia

## Abstract

Parity is a potential confounder of the association between medically assisted reproduction (MAR) and health outcomes. This concept paper describes a population-based record linkage study design for selecting MAR-unexposed women matched to the parity of MAR-exposed women, at the time of the first exposure to MAR. Women exposed to MAR were identified from claims for government subsidies for relevant procedures and prescription medicines, linked to perinatal records. Women unexposed to MAR were identified from linked perinatal and death records, matched to exposed women by age, rurality, age of first child (if any) and parity at the date of first MAR. The availability of a longitudinal, whole-of-population dataset (“population spine”) based on enrolments in Australia’s universal health insurance scheme was a critical design element. The example application examines cancer risk in women after exposure to MAR. Parity is a confounder in this setting because it is associated with MAR and hormone-sensitive cancers.

## Background information

Parity refers to the number of times a woman has given birth to a baby of viable gestation or foetal weight, regardless of the birth outcome (still or live born). In Australia this is 20 weeks’ gestation or 400 grams birthweight. A nulliparous woman is one who has never given birth, although she may have suffered a miscarriage or had a termination of pregnancy before 20 weeks’ gestation. A woman who has given birth once before is primiparous, while a woman who has given birth two or more times before is multiparous.

Parity, as a reproductive factor, can be important to account for in observational studies. It can act as an independent risk factor for obstetric complications including preeclampsia, postpartum haemorrhage, placental abruption, uterine rupture, neonatal mortality and morbidity, and Caesarean section, and often takes on a U-shaped risk profile [1]. Parity can also act as a potential risk factor for longer term outcomes including coronary heart disease, diabetes and some cancers, particularly hormone-sensitive breast and endometrial cancers [2]. A number of biological mechanisms are thought to link parity with non-cancer outcomes, particularly the role of cardiovascular changes during pregnancy. The biological mechanism linking parity with hormonal cancer risk is related to the cumulative exposure to endogenous oestrogen and progesterone during a woman’s life. For example, nulliparity and delayed childbearing are consistently associated with increased cancer risk for oestrogen receptor-positive breast cancer [2]. Therefore, if parity is also associated with an exposure of interest it can act as a potential confounder in epidemiological studies. One such exposure is medically assisted reproduction (MAR) technologies to treat infertility, which generally involve exposure to high levels of exogenous and endogenous hormones during treatment.

The treatment of infertility has been revolutionised over the past 30 years through advances in MAR, with conservatively 5-8% of children conceived following MAR treatment in a number of developed countries [3]. The most common MARs are assisted reproductive technologies (ART) such as in-vitro fertilization (IVF), and ovulation induction (OI) with or without intrauterine insemination (IUI). ART involves a woman’s ovaries being hyper-stimulated using hormones to mature multiple eggs, which are then collected and fertilised outside of the body with partner or donor sperm before being transferred into the uterus. Similarly, OI involves stimulation of the ovaries to induce maturation and ovulation of an egg, coupled with either timed sexual intercourse or IUI of prepared partner or donor sperm. ART has become a mainstream medical procedure with more than 1.5 million ART cycles performed each year globally [4], and a similar number of OI/IUI cycles thought to be performed [5].

The high levels of exogenous and endogenous hormones during MAR treatment is a continuing source of concern for patients and the medical profession, primarily because of the potential carcinogenic effects on hormone responsive tissues such as the breast, the endometrium and the ovary [6]. However, because women who undertake MAR treatment have a different pattern of parity (e.g. older age at first child and lower parity) compared to those who have conceived spontaneously, it is important to account for any differences and the confounding nature of parity in epidemiological studies.

In the following data concept, we describe a method for controlling for parity at the time of first exposure to MAR treatment and at the time of cancer diagnosis in women. The example chosen is based on linked Australian population datasets, but could be generalised to other settings. At the heart of the method is a novel approach for selecting unexposed women matched for parity pattern, age and rurality of residence from a longitudinal, whole-of-population dataset (“population spine”) based on the Australian Medicare Enrolment File (MEF). The MEF contains information on all Australian citizens and permanent residents enrolled in Australia's universal health insurance scheme, Medicare, since 1984.

## Data Sources

Table 1 is a listing of the databases and key variables used to extract the required data for the concept of controlling for parity and other potential confounders of the association between cancer and exposure to MAR in the Australian context. In addition to parity, a woman’s age and residential rurality are potential confounders and thus matching factors. Age is an established, strong risk factor for cancer, and women undertaking MAR are generally older than women who conceive a child spontaneously. With respect to rurality, women living in urban areas are more likely to try to conceive their first child later in life than those in rural areas, and women who live in urban areas also have greater access to MAR than those in rural areas.

**Table 1: Datasets and key variables used to match and account for parity and other potential confounders at the date of first exposure to medically assisted reproduction (MAR):** 

Dataset	Description	Years	Matching variable(s)
Medicare Benefits Schedule (MBS)	A listing of the Medicare services subsidised by the Australian government	1991-2015	Year of birth Date of first MAR service
Pharmaceutical Benefits Scheme (PBS)	A listing of the Medicare prescription drugs subsidised by the Australian government	2002-2015	Year of birth Date of first MAR medication supply
State and territory Perinatal Data Collections (PDC)	A listing of all births, as compiled by each Australian state and territory; records patient characteristics, pregnancy care and outcomes	Variable years, starting from 1980 to 2005, up to 2015	Parity at date of first MAR Woman’s age +/- 365 days at first child (if any) and date of first MAR
Medicare Enrolment File (MEF)	A listing of individuals enrolled in Australia's universal health insurance scheme	1984-2015	Year of birth Rurality of residence
National Death Index (NDI)	A listing of all deaths occurring in Australia	1982-2015	Date of death (Not a matching variable, but needed to ensure MAR-unexposed women alive at the time they are matched to a case's date of first MAR).

## Operationalization of concept

The following is a detailed step-by-step description of the data concept process for constructing MAR-exposed and MAR-unexposed women cohorts.

 Step 1: Identify MAR-exposed women and their parity at date of first MAR (index date)

1. Identify the MAR-exposed women cohorts from the MBS (ART exposure) and the PBS (OI/IUI exposure). Note, Australia is unique in that is subsidises all medically necessary ART through the MBS and PBS and thus enables almost all MAR exposure to be identified using these datasets. Retain year of birth and identify the earliest date of first MAR for all women for each year of birth.
2. Link the MAR-exposed women cohorts to the state and territory Perinatal Data Collections (PDCs) to obtain the parity of each woman (total number of pregnancies of 20 completed weeks or more, or 400gm birthweight at the date of first MAR. If a perinatal record does not exist for a woman before the date of the first MAR, the woman will be assigned a parity of n (nulliparous). If a perinatal record does exist prior to the first MAR date, assign parity (0, 1, 2, 3+) and identify her age at her first birth from the perinatal records. If a perinatal record/s only exists after the first MAR date and the parity is ≥1, assign parity, however the age at first birth will be unknown (this situation arises for women who had children overseas or before the establishment of the PDC).
3. Link with the Medicare Enrolment File to obtain all residential addresses for each woman. Classify residential rurality as urban or rural based on the woman’s residential location >50.0% of the time across her entire Medicare record for the study period using the Accessibility/Remoteness Index of Australia Plus (ARIA+) [7].

Step 2: Identify MAR-unexposed women and their parity at the index date

1. From the Medicare Enrolment File, exclude all men and MAR-exposed women (see Step 1), and classify residential rurality as above.
2. Randomly select MAR-unexposed women:
  a. Restrict to women with a year of birth matching one or more MAR-exposed women. Further restrict to women alive at the earliest date of first MAR for a given year of birth, by linkage with the National Death Index.
  b. Link the file created at Step 2a to the state and territory PDCs to ascertain the parity of each MAR-unexposed woman that coincides with the PDC record (if one more exists) with the closest date preceding the first MAR in the MAR-exposed women. Use the same methods for assigning parity as described above for MAR-exposed women.
  c. Randomly select MAR-unexposed women matched to MAR-exposed women with replacement to minimise bias [8], matching on age (year of birth), rurality, parity (n, 0, 1, 2, 3+) at first MAR, and age at first child if parity ≥1 at first MAR (to account for maternal age at first birth which may confound risk of hormone sensitive cancers).

Matching in our example is 1:4, exposed to unexposed, however depending on the outcome being investigated, different ratios can be applied to maximise efficiency, feasibility and power. The women could also be matched on other potential confounding factors. For studies of health outcomes in MAR-exposed women irrespective of the reproductive outcomes, the scope is limited to variables included in the “population spine”, as these covariates are available for all women. For example, in Australia, women could also be matched on area of residence and socio-economic status. For studies of health outcomes in MAR-exposed women who give birth (e.g. obstetric complications), the women could be matched on variables included in the perinatal records, for example, smoking and BMI.

**Figure 1: Overview of data flows to construct the MAR-exposed women and unexposed (non-MAR) women cohorts:**
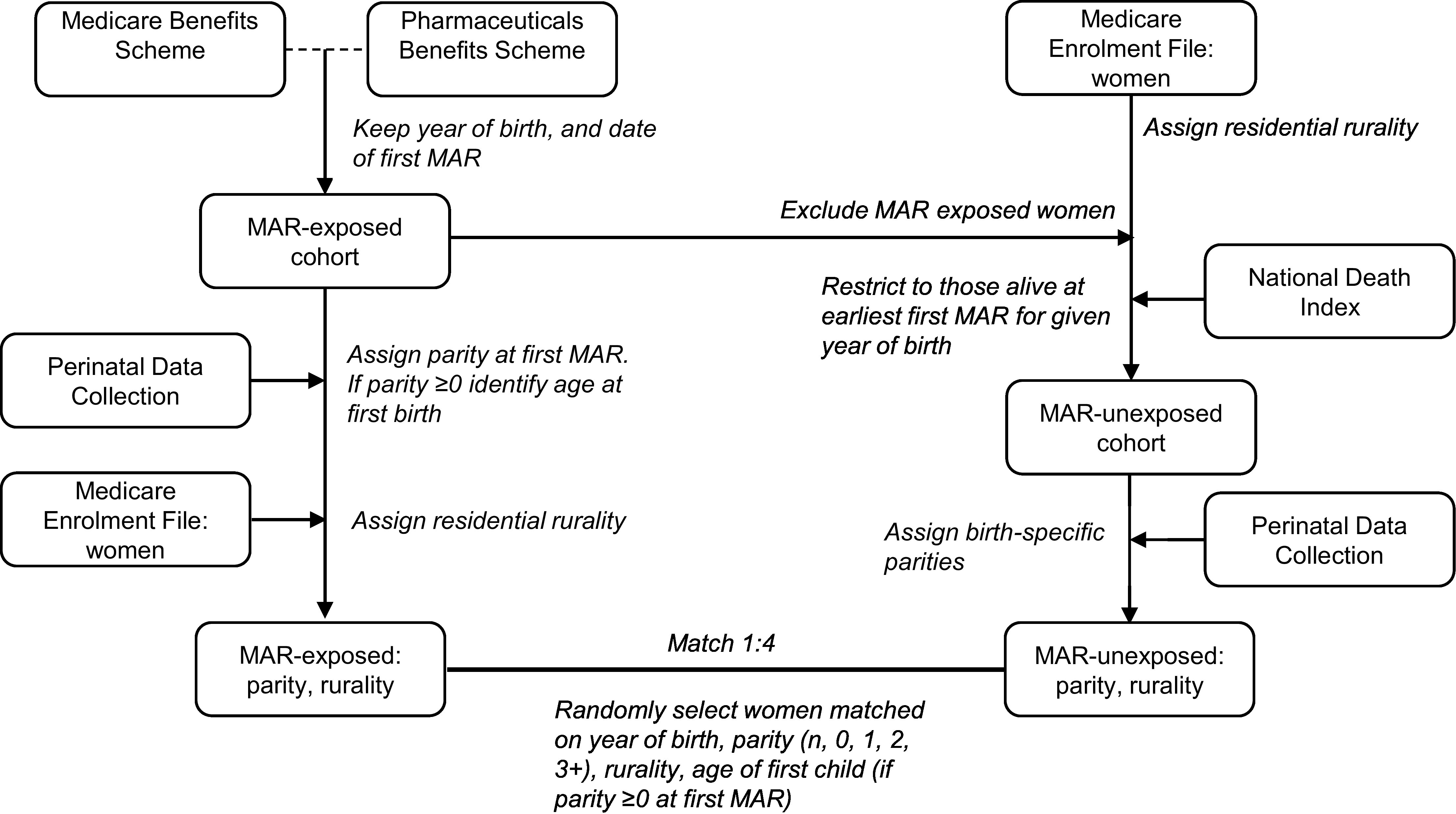


## Analytical approach

Due to systematic differences in parity for MAR-exposed and MAR-unexposed women beyond the time of first MAR exposure, adjustment for cumulative parity at the time of censoring (cancer, death or end of follow-up) is required in addition to matching on parity at the date of exposure. Adjusting for baseline age and parity and other covariates should also be explored [9].

## Alternative sources of parity and MAR exposure

In Australia, PDCs are the gold standard source of parity data. Parity may also be ascertained from vital birth registries. Alternative sources of MAR exposure include clinical quality registries of MAR that exist in most countries [4], including the Australian and New Zealand Assisted Reproductive Technology Database, ANZARD [10], ICD codes describing hospital encounter for MAR treatment (e.g. ICD-10-CM Diagnosis Code Z31.83), health insurance claims data [11], and birth registrations of children born following MAR [12]. ANZARD was not used in this concept because ART cycles were not linked to individual women in ANZARD until 2009.

## Limitations/cautions

Women who gave birth overseas but never in an Australian jurisdiction will be misclassified as nulliparous for both the MAR-exposed and MAR-unexposed cohorts. Migrant women can be identified from their date of registration in the MEF dataset and will be flagged so that we can perform sensitivity analyses to assess the impact of this parity misclassification on cancer risk. Women who only gave birth in Australia before the PDCs started will also be misclassified as nulliparous, and this will be taken into account when interpreting the findings.

## Ethics statement

Ethics approval not required for this data concept.

## References

[1] Williams Obstetrics, Twenty-Fourth Edition Cunningham F, Leveno K, Bloom S, Spong C, Dashe J, Hoffman B, et al., editors. New York, NY: McGraw-Hill; 2013.

[2] Ma H, Bernstein L, MCPike , Ursin G. Reproductive factors and breast cancer risk according to joint estrogen and progesterone receptor status: a meta-analysis of epidemiological studies. Breast Cancer Research. 2006;8(4):R43 10.1186/bcr1525 16859501 PMC1779465

[3] De Geyter C, Calhaz-Jorge C, Kupka MS, Wyns C, Mocanu E, Motrenko T, et al ART in Europe, 2014: results generated from European registries by ESHRE: The European IVF-monitoring Consortium (EIM) for the European Society of Human Reproduction and Embryology (ESHRE), Human Reproduction. 2018;33(9):1586-1601. 10.1093/humrep/dey242 30032255

[4] Adamson GD, de Mouzon J, Chambers GM, Zegers-Hochschild F, Mansour R, Ishihara O, et al International Committee for Monitoring Assisted Reproductive Technologies world report: Assisted Reproductive Technology 2011. Fertility and Sterility. 2018;110(6):1067-1080. 10.1016/j.fertnstert.2018.06.039 30396551

[5] Kulkarni A, Jamieson D, Jones HJ, Kissin D, Gallo M, Macaluso M, et al Fertility Treatments and Multiple Births in the United States. New England Journal of Medicine. 2013;369(23):2218-2225. https://doi.org/10.1056/NEJMoa1301467 24304051 10.1056/NEJMoa1301467

[6] Siristatidis C, Sergentanis T, Kanavidis P, Trivella M, Sotiraki M, Mavromatis I, et al Controlled ovarian hyperstimulation for IVF: impact on ovarian, endometrial and cervical cancer-asystematic review and meta-analysis. Human Reproduction Update. 2013;16(2):105-123 . 10.1093/humupd/dms051 23255514

[7] Hugo Centre for Migration and Population Research, Adelaide Uo. Accessibility/Remoteness Index of Australia (ARIA+). Available at: https://www.adelaide.edu.au/hugo-centre/services/aria. Accessed October 2019

[8] Heide-Jørgensen U AK, Kahlert J, Sørensen HT, Pedersen L. Sampling strategies for selecting general population comparison cohorts. Clinical Epidemiology. 2018;10:1325-1337. 10.2147/CLEP.S164456 30310326 PMC6165733

[9] Sjölander A, Greenland S. Ignoring the matching variables in cohort studies - when is it valid and why? Statistics in Medicine. 2013;32(27):4696-4670. 10.1002/sim.5879 23761197

[10] Fitzgerald O, Paul R, Harris K, Chambers G. Assisted reproductive technology in Australia and New Zealand 2016. Sydney: National Perinatal Epidemiology and Statistics Unit, the University of New South Wales, Sydney 2018.

[11] Lathi RB, Murugappan G, Eisenberg ML, Li S, Baker VL. Risk of cancer in infertile women: analysis of US claims data. 2019. Human Reproduction. 2019; 34(50): 894-902 10.1093/humrep/dez018 30863841

[12] Declercq ER, Belanoff C, Diop H, Gopal D, Hornstein MD, Kotelchuck M, et al Identifying women with indicators of subfertility in a statewide population database: operationalizing the missing link in assisted reproductive technology research. Fertility and Sterility. 2014;101(2):463-471. 10.1016/j.fertnstert.2013.10.028 24289994 PMC3985414

